# Supplementary material for: Salubrinal induces fetal hemoglobin expression via the stress-signaling pathway in human sickle erythroid progenitors and sickle cell disease mice
Source: PLoS One. 2022 May 31;17(5):e0261799. doi: 10.1371/journal.pone.0261799 (PMC9154101; doi:10.1371/journal.pone.0261799)
Supplement: S1 Fig — A) Shown in the bar graph is quantified γ-globin/GAPDH mRNA data generated by RT-qPCR for each treatment conditions after 6-hour incubation and B) after 24-hour incubation. C) Western blot analysis determined HbF levels compared with tubulin as the internal loading control after 6 and 24-hour treatments. D) Quantitative data generated by densitometry analysis shows the expression of protein levels in K562 cells of HbF normalized to tubulin. (DOCX) [file pone.0261799.s002.docx]

**S1 Fig.** Quantitative data generated by densitometry analysis shows the expression of protein levels in K562 cells of **A)** HbF normalized to tubulin, **B)** p-eIF2α normalized to total eIF2α, and **C)** ATF4 normalized to tubulin.  **D**) Shown in the bar graph is quantified γ-globin/GAPDH mRNA data generated by RT-qPCR for each treatment conditions after 6-hour incubation and **E)** after 24-hour incubation**. F)** Western blot analysis determined HbF levels compared with tubulin as the internal loading control after 6 and 24-hour treatments. **G**) Quantitative data generated by densitometry analysis shows the expression levels HbF compared to tubulin.
