# Supplementary material for: Salubrinal induces fetal hemoglobin expression via the stress-signaling pathway in human sickle erythroid progenitors and sickle cell disease mice
Source: PLoS One. 2022 May 31;17(5):e0261799. doi: 10.1371/journal.pone.0261799 (PMC9154101; doi:10.1371/journal.pone.0261799)
Supplement: S2 Fig — Panel A and B show Western blot and quantitative data generated by densitometry analysis shows the expression levels of HbS in sickle erythroid progenitors. The vertical black lines represent the elimination of a water control. Quantitative data generated by densitometry analysis shows the expression of protein levels in sickle erythroid progenitors of C) HbF normalized to β-actin, D) p-eIF2α normalized to total eIF2α, and E) ATF4 normalized to β-actin. (DOCX) [file pone.0261799.s003.docx]

**S2 Fig.** Panel **A and B** show Western blot and quantitative data generated by densitometry analysis shows the expression levels of HbS in sickle erythroid progenitors. The vertical black lines represent the elimination of a water control.  Quantitative data generated by densitometry analysis shows the expression of protein levels in sickle erythroid progenitors of **C)** HbF normalized to β-actin, **D)** p-eIF2α normalized to total eIF2α, and **E)** ATF4 normalized to β-actin.
