# Supplementary material for: Salubrinal induces fetal hemoglobin expression via the stress-signaling pathway in human sickle erythroid progenitors and sickle cell disease mice
Source: PLoS One. 2022 May 31;17(5):e0261799. doi: 10.1371/journal.pone.0261799 (PMC9154101; doi:10.1371/journal.pone.0261799)
Supplement: S3 Fig — A) Schematic showing IP injections administration of SAL, 4–6 months old Townes SCD transgenic mice were treated with 3 or 5 mg/kg of SAL dissolved in water for 4 weeks, water (vehicle) and hydroxyurea (HU; positive control) treatments were completed as controls (N = 10 per group; 5 males and 5 females). (DOCX) [file pone.0261799.s004.docx]

**S3 Fig.** A) Schematic showing IP injections administration of SAL, 4-6 months old Townes SCD transgenic mice were treated with 3 or 5 mg/kg of SAL dissolved in water for 4 weeks, water (vehicle) and hydroxyurea (HU; positive control) treatments were completed as controls (N=10 per group; 5 males and 5 females).
