# Supplementary material for: Salubrinal induces fetal hemoglobin expression via the stress-signaling pathway in human sickle erythroid progenitors and sickle cell disease mice
Source: PLoS One. 2022 May 31;17(5):e0261799. doi: 10.1371/journal.pone.0261799 (PMC9154101; doi:10.1371/journal.pone.0261799)
Supplement: S4 Fig — Peripheral blood was collected in EDTA tubes by tail bleed at week 0, 2, and 4. Blood samples were analysed for automated complete blood counts with differential using a Micros 60 machine (HORIBA Medical/ABX Diagnostics). A-D) WBC, white blood count; Grans, granulocyte; Lymps, lymphocytes; Mono, monocyte; Plts, platelets. In the HU treated group the following had a statistically significant change: WBC (p = 0.0068), Grans (p = 0.041), Lymps (p = 0.023), Mono (p = 0.021), Plts (p = 0.0060 (WK2); 0.0040 (WK4)). Data were generated and shown as the mean ± SEM (n = 10) p<0.05; **p<0.01; *** p<0.001 was considered statistically significant. (DOCX) [file pone.0261799.s005.docx]

**S4 Fig.** Peripheral blood was collected in EDTA tubes by tail bleed at week 0, 2, and 4. Blood samples were analysed for automated complete blood counts with differential using a Micros 60 machine (HORIBA Medical/ABX Diagnostics). A-D) WBC, white blood count; Grans, granulocyte; Lymps, lymphocytes; Mono, monocyte; Plts, platelets. In the HU treated group the following had a statistically significant change: WBC (p=0.0068), Grans (p=0.041), Lymps (p=0.023), Mono (p=0.021), Plts (p=0.0060 (WK2); 0.0040 (WK4)). Data were generated and shown as the mean ± SEM (n=10) p<0.05; **p<0.01; *** p<0.001 was considered statistically significant.
