# Supplementary material for: Salubrinal induces fetal hemoglobin expression via the stress-signaling pathway in human sickle erythroid progenitors and sickle cell disease mice
Source: PLoS One. 2022 May 31;17(5):e0261799. doi: 10.1371/journal.pone.0261799 (PMC9154101; doi:10.1371/journal.pone.0261799)
Supplement: S5 Fig — Peripheral blood was collected in EDTA tubes by tail bleed at week 0, 2, and 4. Blood samples were analysed for automated complete blood counts with differential using a Micros 60 machine (HORIBA Medical/ABX Diagnostics). A-D) RBC, red blood cells; Hb, haemoglobin; HCT, haematocrit; MCV, mean corpuscular volume; MCH, mean corpuscular haemoglobin; RDW, red cell distribution width. In the HU treated group the following had a statistically significant change: HCT (p = 0.052), MCV (p = 0.0028 (WK2); 0.0029 (WK4). Data were generated and shown as the mean ± SEM (n = 10) p<0.05; **p<0.01; *** p<0.001 was considered statistically significant. (DOCX) [file pone.0261799.s006.docx]

**S5 Fig.** Peripheral blood was collected in EDTA tubes by tail bleed at week 0, 2, and 4. Blood samples were analysed for automated complete blood counts with differential using a Micros 60 machine (HORIBA Medical/ABX Diagnostics). A-D) RBC, red blood cells; Hb, haemoglobin; HCT, haematocrit; MCV, mean corpuscular volume; MCH, mean corpuscular haemoglobin; RDW, red cell distribution width. In the HU treated group the following had a statistically significant change: HCT (p=0.052), MCV (p=0.0028 (WK2); 0.0029 (WK4)). Data were generated and shown as the mean ± SEM (n=10) p<0.05; **p<0.01; *** p<0.001 was considered statistically significant.
