# Supplementary material for: Salubrinal induces fetal hemoglobin expression via the stress-signaling pathway in human sickle erythroid progenitors and sickle cell disease mice
Source: PLoS One. 2022 May 31;17(5):e0261799. doi: 10.1371/journal.pone.0261799 (PMC9154101; doi:10.1371/journal.pone.0261799)
Supplement: S1 Raw image — (DOCX) [file pone.0261799.s007.docx]

**S6 Raw data set for Figures 1 through Figure 6.**

**Figure 1**

**Figure 1. cont.**

**Figure 2**

**Figure 3 cont.**

**Figure 3 cont.**

**Figure 4 cont.**

**Figure 5 cont.**

**Figure 6**
