# Supplementary material for: Salubrinal induces fetal hemoglobin expression via the stress-signaling pathway in human sickle erythroid progenitors and sickle cell disease mice
Source: PLoS One. 2022 May 31;17(5):e0261799. doi: 10.1371/journal.pone.0261799 (PMC9154101; doi:10.1371/journal.pone.0261799)
Supplement: S1 File — (DOCX) [file pone.0261799.s008.docx]

**S7 Original Western Blot gels.**
