# Supplementary material for: Salubrinal induces fetal hemoglobin expression via the stress-signaling pathway in human sickle erythroid progenitors and sickle cell disease mice
Source: PLoS One. 2022 May 31;17(5):e0261799. doi: 10.1371/journal.pone.0261799 (PMC9154101; doi:10.1371/journal.pone.0261799)
Supplement: Supplementary file 8 [file pone.0261799.s001.docx]

**S1 Table.** Summary of primer sequences.	
**RT-PCR primers 5'→ 3'**	
Gγ-globin forward	GGCAACCTGTCCTCTGCCTC
Gγ-globin reverse	GAAATGGATTGCCAAAACGG
β-globin forward	CTCATGGCAAGAAAGTGCTCG
β-globin reverse	AATTCTTTGCCAAAGTGATGGG
GAPDH forward	GAAGGTGAAGGTCGGAGT
GAPDH reverse	GAAGATGGTGATGGGATTTC
BCL11A forward	CTCGTTCTGCACATGGAGCTCT
BCL11A reverse	GCAAGAGAAACCATGCACTGGTG
**ChIP assay primers 5'→ 3'**	
ATF4 sites at *-835HBG2* forward	AGAATGGGGCACAGTGGATG
ATF4 sites at *-835HBG2* reverse	CCAGCATCCCGACCATGATT
ATF4 sites at second intron *HBB* forward	GGCCCACAAGTATCACTAAGC
ATF4 sites at second intron *HBB* reverse	CACTGACCTCCCACATTCCC
LCR-HS2 forward	CCTTCTGGCTCAAGCACAGC
LCR-HS2 reverse	ATAGGAGTCATCACTCTAGGC
HBG2 G-CRE forward	CGGCGGCTGGCTAGGGATGAA
HBG2 G-CRE forward	CTGTGAAATGACCCATGGCG
